# Supplementary material for: The interactome of intact mitochondria by cross-linking mass spectrometry provides evidence for coexisting respiratory supercomplexes
Source: Mol Cell Proteomics. 2017 Dec 8;17(2):216–32. doi: 10.1074/mcp.RA117.000470 (PMC5795388; doi:10.1074/mcp.RA117.000470)
Supplement: Supplemental Data [file supp_RA117.000470_133922_0_supp_23978_fzffwf.zip › spectra_annotation/mito_DR_spectra_annotation/207-1-1-1-11-1.pdf]

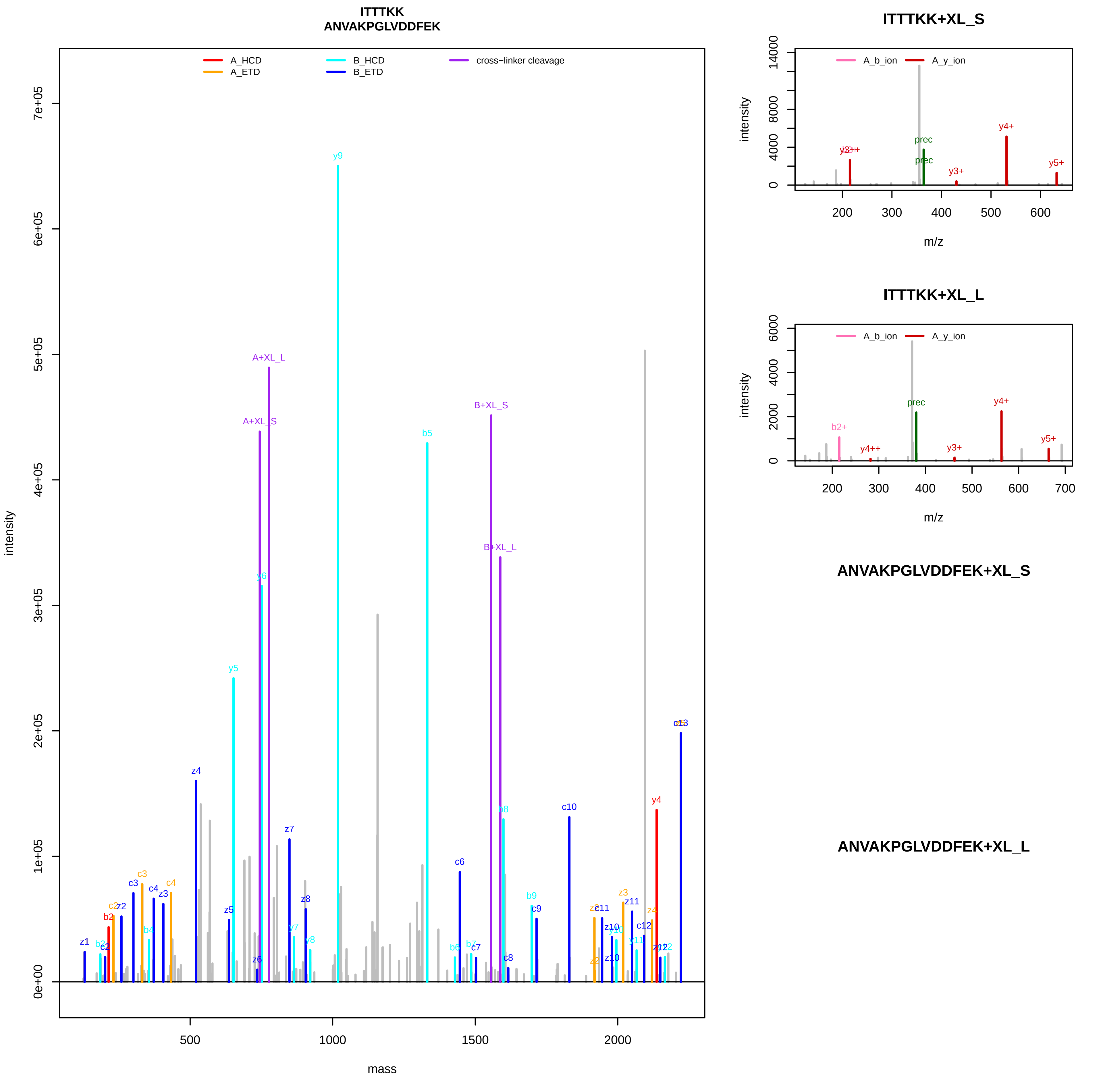

ITTTKK
ANVAKPGLVDDFEK
A_HCD
A_ETD
B_HCD
B_ETD
cross−linker cleavage
intensity
mass
0e+00
1e+05
2e+05
3e+05
4e+05
5e+05
6e+05
7e+05
500
1000
1500
2000
y9
A+XL_L
A+XL_S
B+XL_S
B+XL_L
b5
y6
y5
z4
c13
y4
c10
b8
z7
c6
c3
c4
z3
c3
c4
c2
z2
b2
c2
z1
z5
z8
b9
c9
c11
z3
z11
z4
c12
z12
ITTTKK+XL_S
A_b_ion
A_y_ion
intensity
m/z
prec
y4+
y3+
y5+
y3++
ITTTKK+XL_L
b2+
y4++
ANVAKPGLVDDFEK+XL_S
ANVAKPGLVDDFEK+XL_L
